# MHC-IIB Filament Assembly and Cellular Localization Are Governed by the Rod Net Charge

**DOI:** 10.1371/journal.pone.0001496

**Published:** 2008-01-30

**Authors:** Michael Rosenberg, Ravid Straussman, Ami Ben-Ya'acov, Daniel Ronen, Shoshana Ravid

**Affiliations:** Department of Biochemistry, Faculty of Medicine, The Hebrew University, Jerusalem, Israel; United States Naval Research Laboratory, United States of America

## Abstract

**Background:**

Actin-dependent myosin II molecular motors form an integral part of the cell cytoskeleton. Myosin II molecules contain a long coiled-coil rod that mediates filament assembly required for myosin II to exert its full activity. The exact mechanisms orchestrating filament assembly are not fully understood.

**Methodology/Principal Findings:**

Here we examine mechanisms controlling filament assembly of non-muscle myosin IIB heavy chain (MHC-IIB). We show that *in vitro* the entire C-terminus region of net positive charge, found in myosin II rods, is important for self-assembly of MHC-IIB fragments. In contrast, no particular sequences in the rod region with net negative charge were identified as important for self-assembly, yet a minimal area from this region is necessary. Proper paracrystal formation by MHC-IIB fragments requires the 196aa charge periodicity along the entire coiled-coil region. *In vivo*, in contrast to self-assembly *in vitro*, negatively-charged regions of the coiled-coil were found to play an important role by controlling the intracellular localization of native MHC-IIB. The entire positively-charged region is also important for intracellular localization of native MHC-IIB.

**Conclusions/Significance:**

A correct distribution of positive and negative charges along myosin II rod is a necessary component in proper filament assembly and intracellular localization of MHC-IIB.

## Introduction

Myosin II is a hexamer composed of two heavy chains of ∼200 kDa and two pairs of essential and regulatory light chains. The heavy chains consist of an amino–terminal globular motor domain containing actin-binding and ATPase activities, a neck domain containing binding sites for light chains and a tail domain, which includes α–helical coiled–coil forming rod and a non-helical tailpiece on the C–terminus. The rod domain is responsible for assembly of myosin II monomers into filaments, the functional structures required for myosin II activity. While muscle myosin forms stable filaments, the filaments formed by non-muscle myosin II are transient, participating in dynamic processes like cell motility and cytokinesis [1].

The mechanisms driving filament assembly are not completely understood. A number of studies have demonstrated that the C-terminus region of the coiled-coil of different myosin II subtypes is critical for filament assembly [2]–[5]. Recently, Nakasawa *et al.* proposed a structural model for short C-terminal fragments of non-muscle myosin heavy chain IIB (MHC-IIB). This model predicts that interactions between areas of opposite charge in the C-terminus region potentially drive filament assembly [4]. In addition, myosin rods carry repetitive structural units of 28aa (amino acids), such that the entire helical sequence of the coiled–coil can be mapped as a cyclical pattern of 28aa with alternating positive and negative charges [6]. This charge periodicity dictates axial staggering at multiples of 14aa between adjacent myosin molecules to achieve perfect alignment of the charges along the myosin rods. However, deletions disrupting 28aa periodicity did not significantly affect self-assembly of myosin fragments but altered the pattern of axial repeats in the paracrystals formed by these fragment [7] Thus, charge periodicity may not be the principal driving force for myosin assembly but is important for the assembly of molecules with proper axial staggering [7].

Recent studies in our laboratory provided evidence that the ∼186aa area at the C-terminus region of the coiled-coil in all known human myosin II isoforms is distinct from the rest of the coiled-coil. It carries a net positive charge, whereas the rest of the coiled-coil has a negative charge (Figure S1 and [8]). We also showed that, as well as the 28aa charge periodicity; there is a longer 196aa charge periodicity inside the coiled-coil domain of human myosin II subtypes. This periodicity was suggested to be important for filament formation [8].

Based on this data, we propose the following role for the charged regions in myosin filament assembly. As the filament of myosin molecules grows larger, it is surrounded by a growing “cloud” of net negative charges. A new myosin rod with net negative charge attempting to join this filament must penetrate this negative cloud to find the specific location that best aligns the charges and gives it the lowest free energy. This is achieved by its positive C-terminal end which pulls it to the growing filament, thus allowing it to find the best location for joining the myosin filament. When the primary interaction is established, the joining rod is zipped-up to the filament with a perfect match of charges [8]. According to this model, myosin II filament assembly requires both the C-terminus region with a net positive charge and a 196aa charge periodicity to align the molecules within the filament.

Here we confirm the importance of the rod positively charged C-terminus region for MHC-IIB self-assembly and paracrystal formation *in vitro* and for cytoskeletal association *in vivo* In the negatively charged region no particular sequences were identified as important for self-assembly, yet a minimal area from this region is necessary. Proper paracrystal formation by MHC-IIB fragments requires the 196aa charge periodicity along the entire coiled-coil region. In contrast to self-assembly *in vitro*, negatively-charged regions of the coiled-coil play an important role in the regulation of the cytoskeletal association and intracellular localization of native MHC-IIB. Thus, the positively charged C-terminus region has unique properties compared to the negatively charged sequences. That is, a proper distribution of positive and negative charges along the MHC-IIB rod is necessary for its proper filament assembly and intracellular localization.

## Results

### Deletions in the positively-charged region of Rod impair its assembly *in vitro*

**Figure 1:**
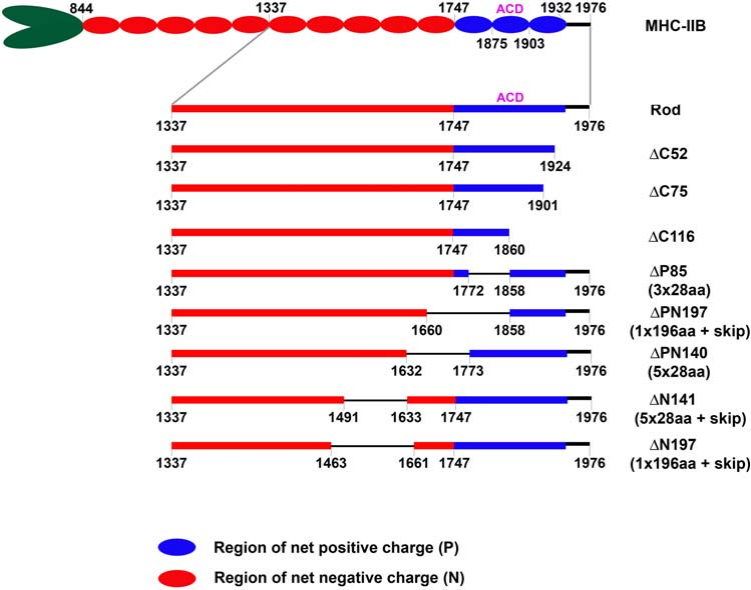
Schematic presentation of MHC-IIB Rod mutants used for in vitro experiments. Rod contains 640aa obtained from MHC-IIB C-terminus. Red and blue boxes indicate regions with net negative (844-1747aa) and positive charge (1748-1931aa), 1932–1976 is the non-helical tail piece. ACD, assembly-competence domain (1875–1903). Where indicated, skip residues were included as extra residues to maintain heptade register. See text for further details.

We have previously shown that in all known human myosin II isoforms, the coiled-coil rod carries a net negative charge except for a 186aa region at the C-terminus that carries a net positive charge (Figure S1, (1747-1932aa) and [8]). The positively charged region contains a short conserved region of 29aa (1875-1903 aa) which is critical for self-assembly of myosin II fragments and is thus termed the “assembly competence domain” (ACD) (Figure 1 and [4], [5]). Together these data may indicate that the entire positively charged region, which includes the ACD is important for myosin II self assembly.

**Figure 2:**
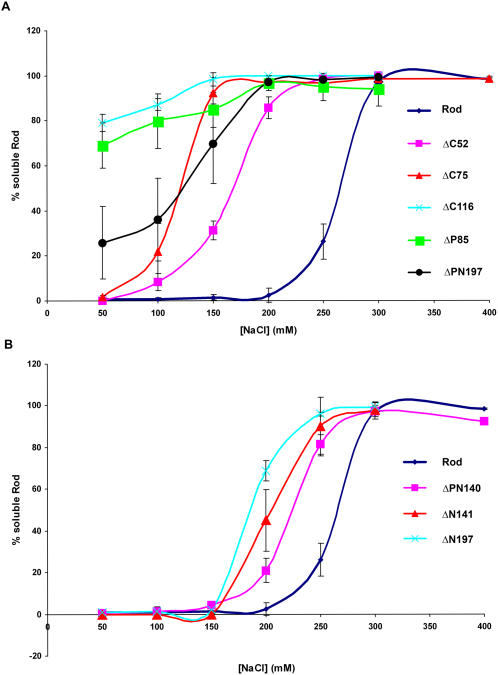
In vitro self-assembly of Rod mutants. *In vitro* self-assembly assays were carried out using 0.5 mg/ml Rod and Rod mutants expressed and purified from *E. coli* as described in Materials and Methods. The proteins were dialyzed against different NaCl concentrations. The extent of self-assembly was calculated as the percentage of mutated Rod proteins remaining in the supernatant after high speed centrifugation. A) Rods with deletions in the region with a net positive charge. B) Rods with deletions from the N-terminus to the region with net positive charge. The results are averages +/− S.D of three to five independent experiments.

To test the importance of this positively charged region for self-assembly we created a fragment comprising 640aa residues from the C-terminus of MHC-IIB, termed Rod, and fragments containing deletions in the positively charged region (Figure 1). We examined the Rod fragments self-assembly properties using several techniques (deletion mutations, solubility assays, charge distribution models, and electron microscopy) that have been used previously to identify regions important for myosin II self-assembly [5], [9]. Solubility assay performed on Rod and Rod mutants (Figure 2A) shows that progressive C-terminal deletions from the positively charged region of the Rod (ΔC52, ΔC75 and ΔC116) resulted in a gradual loss of self-assembly. Deletion of 116aa, which included the ACD, rendered the Rod virtually unable to assemble in the entire range of salt concentrations tested.

To determine whether other positively charged regions upstream to the ACD are also important for self-assembly, we created a ΔP85 mutant that preserved 28aa charge periodicity (Figure 1). Surprisingly, although ΔP85 contains the ACD, self-assembly was dramatically decreased and was similar to that of ΔC116 mutant (Figure 2A). Hence it seems that while ACD is important it is not sufficient for self–assembly; the entire positively charged region is necessary for efficient self-assembly.

### The role of the negatively charged regions in Rod self-assembly

It was previously shown that self-association of Dictyostelium discoideum myosin II fragments requires a minimum size of 294aa that contains two positive and two negative clusters, and that the interaction between clusters of opposite charge mediates self-assembly [10]. Similar phenomenon was recently observed by Nakasawa *et al.* [4]. They demonstrated that MHC-IIB fragments required minimal length in order to self-assembly. This suggests that negative charge areas may play an important role in MHC-IIB self-assembly. To confirm that a negatively charged region is required for self-assembly we prepared two new deletions, ΔPN140 and ΔN141 (Figure 1, ΔPN140 mutant contains 26aa of the positively-charged region). These deletions were designed to conserve the 28aa charge periodicity. *In vitro* solubility assays showed that these deletions had a relatively minor negative effect on the assembly properties of the Rods (Figure 2B) compared to the deletions made in the positively-charged region (Figure 2A). Hence, our results indicate that a negatively-charged coiled-coil region is indeed required to interact with the positively-charged region, however this region is not restricted to a specific zone of the negatively charged region.

### 196aa charge periodicity is important for correct paracrystal formation *in vitro*

It was previously suggested that preserving 28aa periodicity is sufficient for self-assembly but not for the formation of paracrystals with proper axial repeat [7]. Instead, 196aa charge periodicity was thought to determine the packing of the myosin molecules inside the filament [6], [8], [9]. To test this hypothesis we created two deletions inside the Rod (ΔPN197 and ΔN197) with preserved 196aa periodicity and intact ACD region.

Surprisingly, ΔPN197 mutant had a higher ability to assemble than ΔP85 mutant, despite the fact that the fewer amino acids were deleted in ΔP85 than in ΔPN197 (85aa and 197aa respectively) (Figure 2A). ΔN197 mutant was less able to self-assemble than Rod, ΔPN140 and ΔN141, most probably due to its being shorter (Figure 2B).

**Figure 3:**
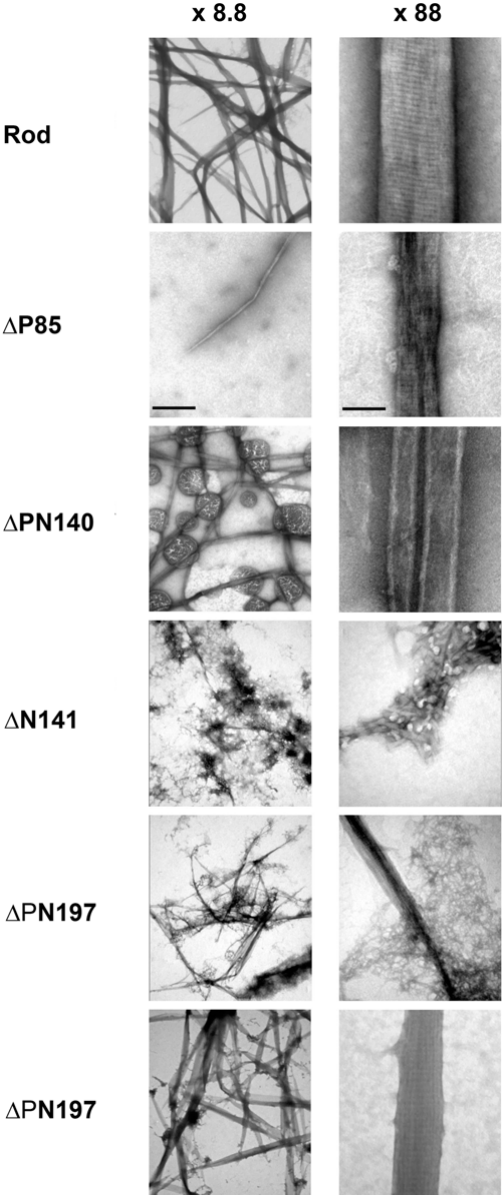
Electron micrographs of paracrystals formed by Rod mutants. Rods were dialyzed for 16 hrs against low salt buffer, stained with uranyl acetate and observed with the electron microscope. Bar on the left column, 1 µM. Bar on the right column, 0.1 µM.

Electron microscopy (see Materials and Methods) revealed that Rod created paracrystals with the 14.5nm striation pattern expected for myosin II (Figure 3 and [11]). ΔP85 created very short and narrow filamentous structures with a very similar striation pattern. ΔPN140 created very long tube-like structures with no apparent striation, while ΔN141 created very short longitudinal structures; most were without striation but a small percentage showed aberrant striation. Although ΔPN197 and ΔN197 preserve 196aa charge periodicity, the former created narrow or aberrant paracrystals, while the latter was the only mutant that formed paracrystals similar in appearance to those of Rod and with the same striation pattern (Figure 2). The difference between these two mutants is that ΔPN197 lacks part of the positively charged region while this area is intact in ΔN197. These results indicate that preservation of 196aa charge periodicity and an intact positive charge region are important for the formation of normal paracrystals.

### Distribution of charged zones in Rod mutants determine paracrystal formation

**Figure 4:**
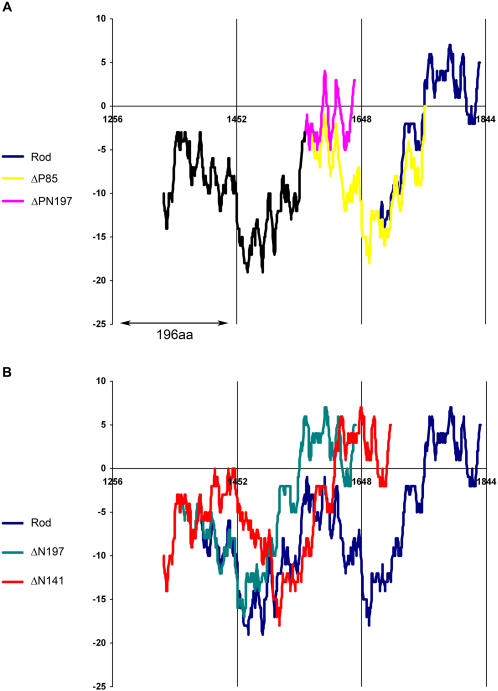
Distribution of the net charge along Rod mutants. The net charge along the coiled-coil region of Rod mutants was estimated using the “sliding window” technique (see Materials and Methods). The resulting data was plotted as the net charge of the window against the first residue of the window. A) Rod mutants with deletions in the positively-charged region. Black region represents the overlap of the charge distribution among all three constructs from residue 1337 to 1559. Note the overlap between Rod and ΔP85 between residues 1560–1679. B) Rod mutants with mutations in the negatively-charged region. Note the overlap of the charge distribution among all constructs between residues 1337–1367.

It has been assumed that preservation of the 196aa periodicity is necessary for the formation of paracrystals with the appropriate axial repeat [6], [8]. However, as described above, ΔPN197 and ΔN197 mutants that preserve 196aa periodicity formed very different paracrystals. Since ΔPN197 lacks part of the positively charged region, we speculated that this mutant has a different distribution of positively and negatively charged zones, leading to the aberrant paracrystals. To examine this hypothesis, we used the “sliding window” technique (see Materials and Methods) to investigate the effect of each deletion on the distribution of the net charge along the coiled-coil region. As shown in Figure 4, the positioning of the zone of net positive charge in Rod and ΔN197 preserved their 196aa periodicity. In contrast, in ΔPN197 the positively charged zone was shifted and the 196aa periodicity was not preserved (Figure 4A).

Using the same technique we examined the charge distribution of Rod mutants preserving 28aa periodicity. Although ΔP85 lacks the positively charged region, its charge distribution is similar to that of Rod (Figure 4A) and it creates small paracrystals with proper striation pattern (Figure 3). ΔPN140 and ΔN141 showed similar shifts in the location of positively charged zone, which would explain their inability to create assemblies with proper axial stagger (Figure 4B and data not shown for ΔPN140). These results indicate that a correct charge distribution is required for the formation of paracrystals with the appropriate axial repeat.

The sliding window technique also allowed us to explain why ΔP85, unlike ΔPN197, was almost unable to self-assemble, although it has a shorter deletion (Figure 1, and Figure 2). The partial deletion in the positively charged region of ΔP85 disrupts the balance between the positive and the negative charges more severely than in ΔPN197 (Figure 4A). It results in complete elimination of the net positively charged region. In contrast, ΔPN197 preserves a small zone of positive charge (Figure 4A), that can to a certain extent support self-assembly. These differences in charge distribution are reflected by the size of the paracrystals formed by these mutants. ΔP85 forms short paracrystals, while ΔPN197 formed much longer paracrystals (Figure 3). These results indicate that a correct charge distribution is required for proper self-assembly.

Analysis of the parallel interactions between adjacent myosin rods revealed a staggering of 14.3 nm and 43 nm, corresponding roughly to the 98aa and 295aa (3×98aa+skip residue). These staggerings are considered thermodynamically favorable [6], [8], [12]. Indeed it was shown that they are important for paracrystal formation [13], [14]. We found here that deletions that preserved 28aa periodicity (ΔN141) did not grow paracrystals with the 14.5nm axial repeat as Rod did. However, deletion in the same place but which preserved 196aa charge periodicity (ΔN197) formed normal paracrystals. We speculate that staggering at 98aa and 295aa may become unfavorable if the 196aa charge periodicity is not preserved.

**Figure 5:**
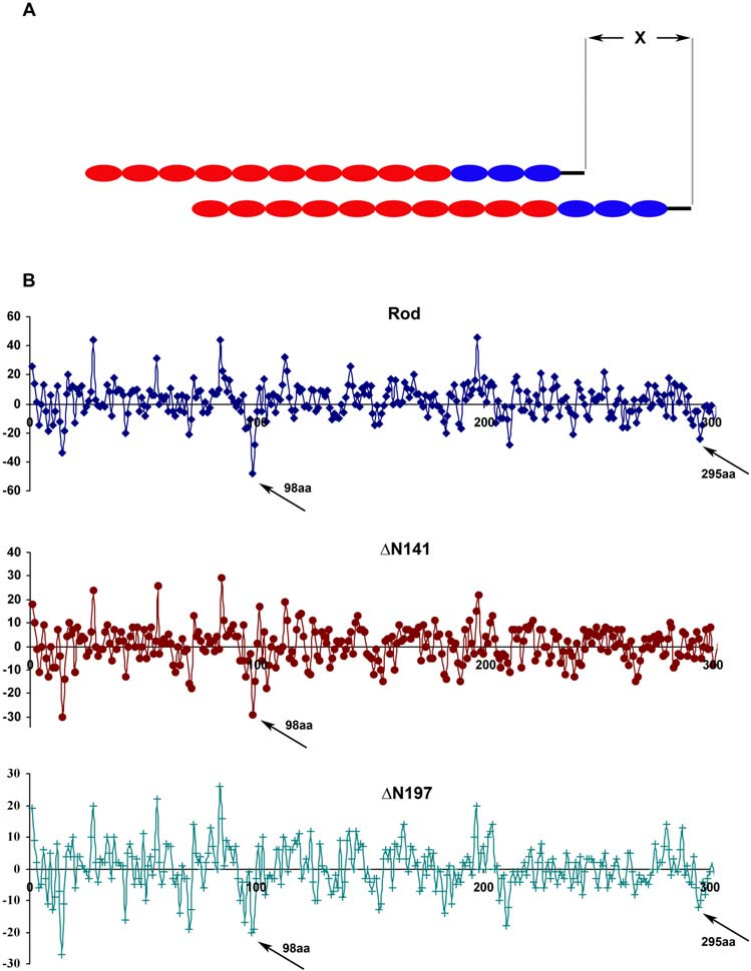
Charge interactions between the mutated Rods. A) Schema of two Rod molecules associated in a parallel orientation. X = staggering. B) Charge interactions between parallel rods. Staggering (X) is plotted horizontally and the net sum of attractions minus repulsions for each X is plotted vertically. Attraction is represented by −1 and repulsion by +1. Skip residues are included in the linear sequence. All *a* and *d* positions of the heptads repeat were changed to zeros. Arrows indicate strong net negative (attractive) interactions, at 98aa and 295aa staggering. The data for staggers higher than 300aa was omitted for clarity.

We therefore analyzed the parallel interactions between the coiled-coil regions of Rod, ΔN141 and ΔN197 (see Materials and Methods). Figure 5 shows a peak of attraction at 98aa staggering for all three proteins. However, only Rod and ΔN197 had a peak of attraction at staggering at 295aa. This may explain the inability of ΔN141 mutant to create paracrystals with 14.5nm axial repeat. The same analysis of ΔPN197 revealed two peaks of attraction similar to Rod and ΔN197 (data not shown), yet this Rod mutant was unable to create paracrystals with 14.5nm axial repeat (Figure 3). Deletion in the positively charged region in ΔPN197 possibly resulted in this phenotype. These results suggest that two basic conditions must be satisfied for formation of paracrystals with the appropriate axial staggering: 196aa periodicity of charges along the entire coiled-coil region and an intact net positively charged region.

### Deletions affect the association of MHC-IIB with the cytoskeleton *in vivo*

**Figure 6:**
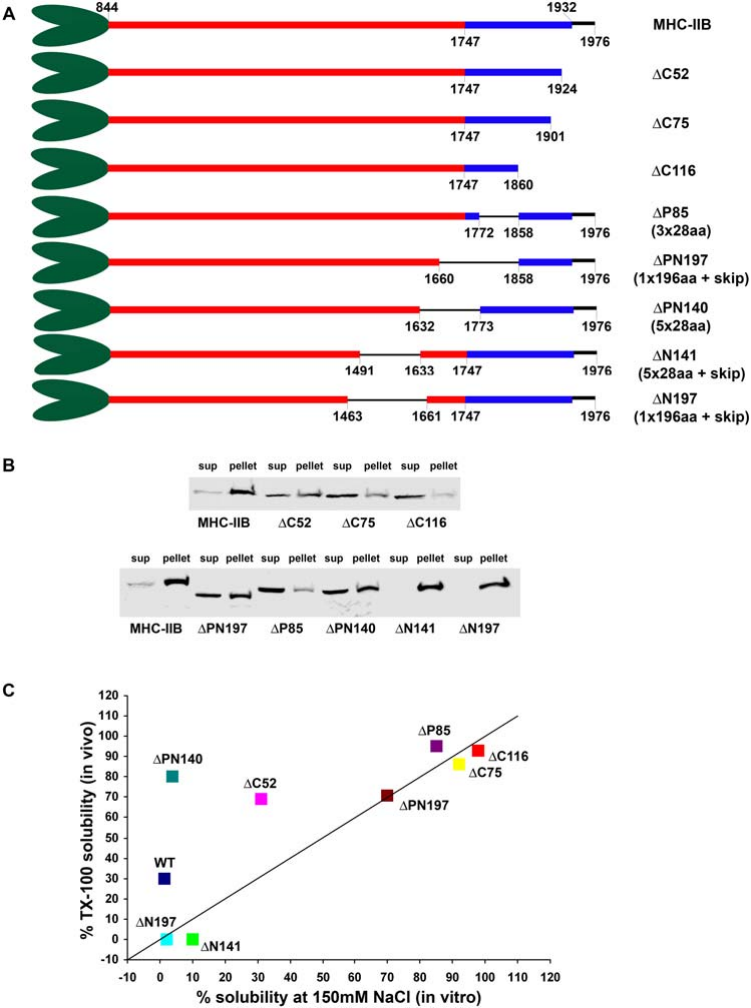
The effect of deletion on cytoskeletal association of MHC-IIB. A) Schematic presentation of MHC-IIB mutants used in *in vivo* experiments. Red and blue boxes indicate regions with net negative (844-1747aa) and net positive charge (1748-1931aa). 1932–1976 is the non-helical tail piece. Where indicated, skip residues were included as extra residues to maintain heptade register. See text for further details. B) B^−^/B^−^ MEF cells were transiently transfected with MHC-IIB or MHC-IIB mutants fused to GFP (see Materials and Methods). Cells were subjected to a TX-100 solubility assay and the percentage of total MHC-IIB in the soluble fraction was determined (see Materials and Methods). Shown are representative experiments of TX-100 solubility of MHC-IIB mutants with progressive C-terminus deletions (upper panel) and deletions upstream to Δ116 (lower panel). Note that the amount of expressed protein is similar. C) Comparison of self-assembly at physiological salt concentration and cytoskeletal association between Rods and native MHC-IIB carrying the same mutations.

Myosin II filament assembly is important for its cellular localization [3], [5]. Therefore, we examined whether the regions we identified as important for self-assembly and paracrystal formation also play a role in cellular localization of MHC-IIB. To do this, we constructed a full length MHC-IIB fused to GFP on its N-terminus, with the same deletions as the Rod mutants (Figure 6A). The MHC-IIB mutated proteins were expressed in mouse embryonic fibroblasts obtained from MHC-IIB knockout mice (MEF B^−^/B^−^) [15]–[17]. Meshel et al. [17] has shown that MHC-IIB fused to GFP expressed in these cells is similarly localized to endogenous MHC-IIB in wild-type fibroblasts. The ability of mutated MHC-IIB to associate with the cytoskeleton was tested using the Triton X-100 (TX-100) solubility assay (see Materials and Methods).

A representative TX-100 solubility assay is shown in Figure 6B. Self-assembly *in vitro* and cytoskeletal association *in vivo* of the various deletion mutants are plotted on the same graph to identify which regions are important both *in vitro* and *in vivo* (Figure 6C). *In vitro*, Rod is completely insoluble at physiological salt concentration (150 mM NaCl), whereas ∼30% of MHC-IIB entered the TX-100 soluble fraction (presumably not a cytoskeletal fraction). This may reflect additional levels of regulation apart from filament assembly *per se* (Figure 6C and Figure S2). ΔC75, ΔC116, ΔP85 and ΔPN197 (deletions in the positively charged region) show similar reductions of both self-assembly and cytoskeletal association. This indicates that the positively charged region plays an important role both *in vitro* and *in vivo*. ΔN141 and ΔN197 were completely insoluble at physiological salt concentration and in TX-100 (Figure 6 and Figure S2). These deletions appear to disrupt regulation elements other than self-assembly that exist in MHC-IIB and are required for normal cytoskeletal association. ΔC52 and ΔPN140 are significantly more soluble in TX-100 than in physiological salt solution. Hence, the regions deleted in ΔC52 and ΔPN140 are important for the association of the whole MHC–IIB molecule with the cytoskeleton.

In summary, these results indicate that MHC-IIB has regions that positively affect its association with the cytoskeleton, while other regions have a negative effect. The interplay between these regions allows the MHC-IIB to associate and disassociate from the cytoskeleton, this being the hallmark of MHC-IIB function.

### Self-assembly is essential for proper MHC-IIB cellular localization

**Figure 7:**
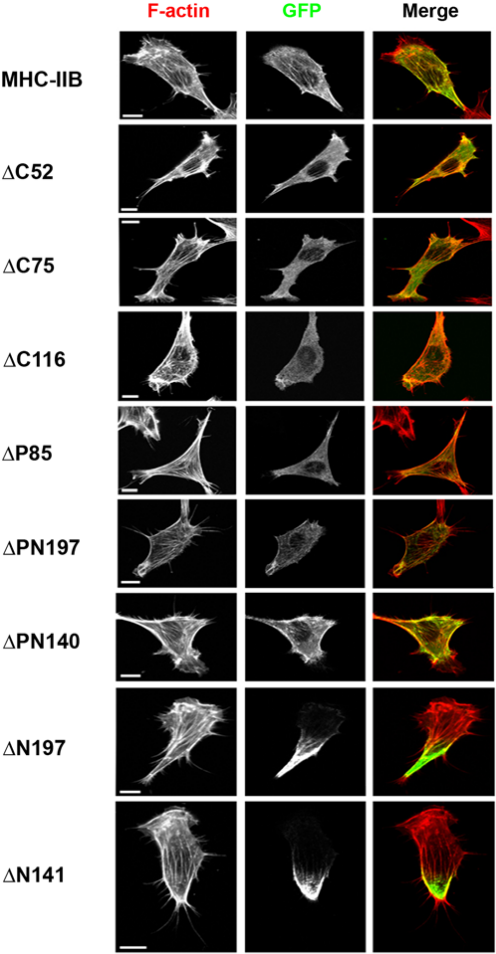
The effect of deletion on intracellular localization of MHC-IIB. B^−^/B^−^ MEF cells were transiently transfected with MHC-IIB or MHC-IIB mutants fused to GFP (see Materials and Materials and Methods and Figure 5A). After transfection cells were serum–starved, fixed and stained for filamentous actin with Rhodamine phalloidin. Representative cells demonstrating the localization properties of MHC-IIB mutants are shown. Red, F-actin; green, MHC-IIB fused to GFP. Note the localization of proteins to stress fibers and the cortical region. Bar = 10 µM.

The regions identified as important for cytoskeletal association of MHC-IIB were further examined for their role in the cellular localization of MHC-IIB. Figure 7 shows that MHC-IIB localized at the cell cortex, at stress fibers and at the lamellar region where bipolar myosin II filaments have been observed in cultured cells [18]–[21]. Deletions in the positively charged region led to a diffuse appearance of MHC-IIB mutants (i.e. ΔC52, ΔC75, ΔC116, ΔP85, ΔPN140 and ΔPN197). While ΔC52 and ΔC75 mutants can be seen in stress fibers and cell cortex, as well as diffusely distributed throughout the cytosolic region, Δ116 and ΔP85 mutants diffused throughout the cytosol (Figure 7). Although ΔPN197 and ΔPN140 mutants appeared more diffuse than wild-type MHC-IIB, they were capable of entering cytoskeletal structures such as stress fibers and cell cortex (Figure 7). However, ΔN141 and ΔN197 showed a stronger tendency to accumulate at the posterior end of polarized dispersed cells than MHC-IIB (Figure 7). This accumulation was characteristic to polarized migrating cells as accessed by wound-scratch assay (Figure S3A), but not for stationary cells where ΔN197 mutant was evenly distributed in stress fibers throughout the cell (Figure S3B). Furthermore, this accumulation was not followed by F-actin accumulation. Co-localization of ΔN197 and actin was observed only in the cortical area of the posterior end of the cell (Figure 7). ΔN197 and ΔN141 appeared to emerge from the accumulation area at the posterior end of the cell and enter stress fibers, cell cortex and the lamellar region (Figure 7). Thus there appears to be a correlation between TX-100 solubility and the ability of these proteins to localize at cytoskeletal structures. MHC-IIB mutant proteins with a higher TX-100 solubility than MHC-IIB had a diffuse cellular appearance, while TX-100 insoluble MHC-IIB mutants accumulated at the posterior part of the cell. We conclude that the positively and the negatively charged regions must be intact for proper cellular localization of MHC-IIB into the cytoskeleton.

## Discussion

Myosin II exerts its activity both in muscle and non-muscle cells when it is in filamentous state and associated with actin filaments [22], [23]. Two regions in the C-terminus of the MHC-IIB coiled-coil, nACD1 and nACD2, have been shown to be critical for filament assembly *in vitro* [4]. The nACD2 region is homologous to the 29aa region identified as ACD, which is critical for the assembly of C-terminus fragments of skeletal muscle myosin *in vitro* [5]. The extended region of 63aa around this 29aa region is conserved in both vertebrate and invertebrate myosin II rods and also in paramyosin rods [24].

The above researchers noticed that the extended ACD region has a unique charge profile, a relatively neutral net charge with a higher percentage of large apolar surface residues than the rest of the coiled-coil. In contrast, most of the myosin II rod carries a net negative charge. Our analyses of successive 98aa regions of the myosin II rods confirmed that all human myosin II subtypes carry a net negative charge along most of the coiled–coil domain, except for the C-terminus where the net charge is positive [8]. Here we have shown that this net positively charged C–terminus region, which includes the ACD, is critical for MHC-IIB assembly *in vitro* and for association with the cytoskeleton *in vivo*. Deletions inside this region, which severely affected the balance between positive and negative residues, impaired self-assembly of Rod fragments *in vitro* and localization of MHC-IIB into the cytoskeleton *in vivo*.

In filament assembly this region appears to create a special area in the rod where positive charge predominates. This area is thus able to interact with the negatively charged areas on another monomer to form a filament. Another role that we propose for this region is to allow a new monomer to join a growing filament by helping it penetrate the “cloud” of negative charges formed by negatively-charged areas of the monomers that had already joined the growing filament. The ACD region alone confers some assembly properties on myosin II monomers, since ΔP85 mutant, whose ACD domain is intact but whose entire zone of positive charge is missing (Figure 4A), can still self–assemble to a certain extent (Figure 2A). However, the formation of larger aggregates may be impaired by the lack of the net positive charge. Additional monomers would be unable to join the growing filament because of the cloud of negative charges surrounding it.

In this context, Sohn *et al.* were able to create assembly–competent protein by fusing the ACD of skeletal muscle myosin to an N–terminal assembly–incompetent fragment [5], which is thought to carry net negative charge [8]. The solubility properties of this fusion protein were similar to the ΔP85 mutant, suggesting that intact ACD region may potentially mediate interactions with other monomer/s by electrostatic interactions [4] and/or by other kind of interactions like apolar interactions proposed for striated muscle myosin II assembly in nematode [25]. However, ACD is insufficient for proper MHC-IIB self-assembly; an intact positively-charged region is also required.

**Figure 8:**
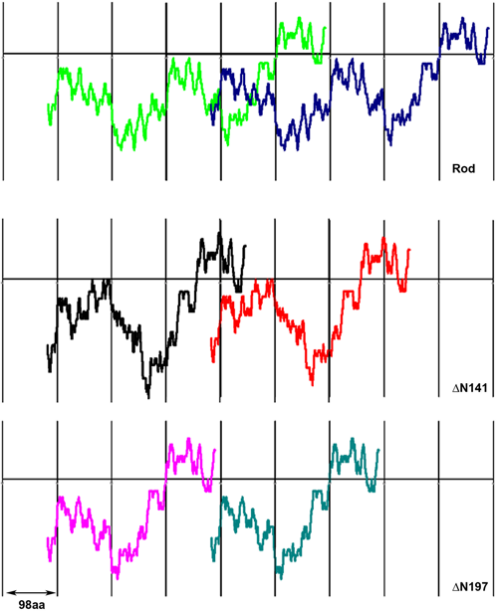
Schematic representation of charge alignment during parallel association with 295aa staggering in Rod and Rod mutants with and without preserving 196aa periodicity. Different colors are used in each plot for the same constructs for clarity. Note that in Rod and Rod with ΔN197 deletion (196aa periodicity preserved), the positively charged zone exactly faces a zone with high net negative charge. However, in Rod with ΔN141 deletion (28aa periodicity preserved), only part of the positively charged zone faces the region with the high negative charge.

Another issue examined in this study is the role of 196aa charge periodicity in the coiled-coil region of myosin II. Atkinson & Stewart [7] found in skeletal muscle myosin C-terminus fragment that deletions upstream to the region responsible for filament assembly, which preserved only 28aa charge periodicity, resulted in abnormal paracrystal formation, without affecting self-assembly into aggregates. They predicted a charge periodicity longer than 28aa in the myosin II rod which controls the alignment of myosin II molecules inside the filament. Our data shows that the 196aa periodicity must be preserved along the entire coiled-coil region for proper paracrystal formation. This distribution predicts that axial staggering between neighboring myosin II molecules occurs at multiples of 98aa. As this conforms to both the 196aa and 28aa charge periodicity (98aa = 7×14aa), this staggering should provide the best alignment of charges. Figure 5 shows that ΔN141, which did not preserve 196aa periodicity, does not have high attraction score at 295aa staggering, unlike Rod and ΔN197 in which 196aa periodicity was preserved. When two adjacent monomers of Rod or ΔN197 are positioned with 295aa staggering between them, the region of the net positive charge is aligned with the high negative charge region (Figure 8). In contrast, when two adjacent monomers of ΔN141 which preserves only 28aa periodicity, are positioned with 295aa staggering between them, only part of the positive region is aligned with the high negative charge area (Figure 8). These monomers will most likely be positioned with a different staggering to achieve a more favorable interaction. Since 295 aa staggering, corresponding to 43nm, is important for the formation of paracrystals with 14.5 nm axial repeat [13], [14], this explains the inability of ΔN141 to form normal paracrystals.

One of the important issues in this study is whether self-assembly and paracrystal formation by MHC-IIB fragments *in vitro* is the correct model for filament formation by intact MHC-IIB *in vivo*. Association of intact MHC-IIB with the cytoskeleton may be influenced by interaction of the motor domain with the cytoskeleton [26], by light chain phosphorylation [27], heavy chain phosphorylation [28], [29] and by interaction with other cytoskeletal proteins [30]–[32]. Nevertheless, regarding filament assembly as the basic property of myosin II isoforms including MHC-IIB, we hypothesized that the regions critical for self-assembly of MHC-IIB fragments are also important for cytoskeletal localization of MHC-IIB *in vivo.* Indeed mutations in the region with the net positive charge, which impaired self-assembly of Rod fragments *in vitro,* also impaired association of MHC-IIB with the cytoskeleton *in vivo* (Figure 6C).

We also found that Rod was completely insoluble in physiological salt concentrations *in vitro*, while MHC-IIB was ∼30% TX-100-soluble *in vivo*. This solubility is possibly mediated by negatively charged regions at the coiled-coil, since the mutants ΔN141 and ΔN197, which lack parts of these regions, are TX-100-insoluble. It is well known that *in vitro* non–muscle and smooth muscle myosin II form a folded conformation resulting in filament disassembly, unless phosphorylated on the regulatory light chains [33], [34]. Kolega & Kumar [35] found that in endothelial cells, TX-100–insoluble myosin II shows a 5 times greater regulatory light chain phosphorylation than TX-100–soluble myosin II. It is possible that ΔN141 and ΔN197 lack the region required for folding and thus these molecules are inherently unfolded, leading to complete TX-100-insolubility. A similar region was identified in smooth muscle myosin II, which contains the interaction site with the regulatory light chains in the folded conformation [36].

ΔN141 and ΔN197 mutations caused a marked accumulation of MHC-IIB in the posterior end of polarized cells. It was shown that localization of MHC-IIB to the posterior end of migrating cells is mediated by filament assembly and light chain phosphorylation through the action of Rho-dependent kinase [27], [37]. Thus, the inability of ΔN141 and ΔN197 to adopt a folded conformation may result in unregulated filament assembly *in vivo* with a subsequent accumulation in the cell posterior end of polarized cells. Nevertheless, the possible interaction of intact MHC-IIB with regulatory proteins *in vivo* and/or other mechanisms cannot be ruled out.

Other exceptions, where self-assembly *in vitro* did not correlate well with cytoskeletal association *in vivo,* were ΔC52 and ΔPN140 mutations (Figure 6C). ΔC52 mutation had a far greater negative effect on TX-100 solubility of MHC-IIB than on self-assembly of Rod *in vitro* (Figure 2A). ΔPN140 had a prominent negative effect on the association of the intact MHC-IIB with the cytoskeleton but a very minor negative effect on the self-assembly of Rod *in vitro*. Sato *et al.* [38] identified two regions on MHC-IIB responsible for the self-recognition between MHC-IIB molecules during the formation of the homo-filament *in vivo*: N57 (1672-1728) and C63 (1914–1976). The N57 region was completely eliminated in ΔPN140 mutant (Figure 1), and the C57 region was partially deleted in ΔC52 mutant (Figure 1). Therefore, it seems that, *in vivo*, self-recognition, using some unidentified mechanism, is also important for cytoskeletal association of the MHC-IIB.

In summary, in this study we explored the mechanisms of self-assembly and paracrystal formation by MHC-IIB C-terminal fragment *in vitro* and cytoskeletal association of the native MHC-IIB *in vivo* (summarized in Tables 1 and 2). While additional mechanisms other than self-assembly seem to be involved in cytoskeletal association of MHC-IIB, the regions which are critical for self-assembly *in vitro* are also critical for cytoskeletal association *in vivo*. The precise distribution of the net charge along the myosin II rod seems to be the basic property for guiding self-association of myosin II molecules into ordered filaments, the basic unit for myosin II function.

**Table 1:** Summary of *in vitro* experiments.

	ACD	Deletion in the area with net negative charge	Net positive charge area	196aa charge periodicity along the coiled-coil	NaCl concentration with 50% solubility (mM)	Paracrystal morphology	Axial repeat (nm)
**Rod**	**Intact**	-	**Intact**	+	270	Normal	14.5
**ΔC52**	**Intact**	-	**Partially disrupted**	+	165	NT	NT
**ΔC75**	**Partially deleted**	-	**Partially disrupted**	+	120	Abnormal*	14.5*
**ΔC116**	**Completely deleted**	-	**Partially disrupted**	+	<50 mM	NT	NT
**ΔP85**	**Intact**	+	**Completely eliminated**	+	<50 mM	Abnormal	14.5
**ΔPN197**	**Intact**	+	**Partially disrupted**	-	120	Abnormal	Undefined
**ΔPN140**	**Intact**	+	**Minor disruption**	-	240	Abnormal	Undefined
**ΔN141**	**Intact**	+	**Intact**	-	250	Abnormal	Undefined
**ΔN197**	**Intact**	+	**Intact**	+	175	Normal	14.5

* - data not shown for the ΔC75; NT–not tested

**Table 2:** Minimal requirements for the processes investigated in this study

	Self-assembly of Rod in vitro	Association of the MHC-IIB with the cytoskeleton
**ACD**	+	+
**Intact area with net positive charge**	+	+
**Minimal area with net negative charge**	+	+
**N57 region**	-	+
**C63 region**	+	+

## Materials and Methods

### Cell line and culture conditions

MHC-IIB knockout mouse embryonic fibroblast cell line (B^−^/B^−^ MEFs) was a generous gift of Dr. R.S. Adelstein (Laboratory of Molecular Cardiology and Laboratory of Molecular Physiology, National Institutes of Health, Bethesda). Cells were maintained in high glucose DMEM supplemented with 2 mM L–glutamine, 10% fetal calf serum and antibiotics (100 units/ml penicillin, 100 µg/ml streptomycin and 1∶100 Biomyc3 anti-mycoplasma antibiotic solution, Biological Industries, Beit HaEmek, Israel). They were grown at 37°C in a humidified atmosphere of 5% CO_2_ and 95% air.

### MHC-IIB used for this study

Accession number, A59252; coiled-coil region, 844-1931; skip residues, 1193, 1586, 1811.

### Construction of MHC-IIB mutants

To create mutated Rods, we used MHC-IIB642 in pET21C vector (pET21C-MHC-IIB642) that encodes for 640 residues from MHC-IIB C-terminus (Figure 1; Rod, (1337–1976)) with the addition of two amino acids on the N-terminus from the vector [39]. The Rod was subjected to a series of mutagenesis reactions using QuikChange™ Site Directed Mutagenesis Kit (Stratagene, La Jolla, CA) according to the manufacturer's instructions. The mutations were confirmed by DNA sequencing (Center for Genomic Analysis, The Hebrew University, Jerusalem). Deletions were created by introducing KpnI restriction sites at both sides of the desired deletion. After digestion and self-ligation, the KpnI site was corrected according to the original sequence using the site directed mutagenesis kit. To create mutated MHC-IIB fused to GFP (GFP-MHC-IIB), the mutated pET21C-MHC-IIB642 were digested with SmaI (Fermentas, Vilnius, Lithuania) and the resulting fragments were cloned into SmaI digested plasmid vector pEGFP-C3 (Clontech, San Jose, CA) containing the entire coding sequence of MHC-IIB (pEGFP-C3–MHC-IIB) (kindly provided by Dr. R.S. Adelstein). All the deletions inside the coiled-coil were multiplications of 28aa-residue unit. Skip residues, which interrupt the heptade repeats, were included in the deletions as extra residues.

### Purification of MHC-IIB fragments from E. coli, solubility assay and negative staining

Purification, solubility assay and negative staining were performed as described previously [39].

### Transient transfection

Cells were plated on 30 mm tissue culture dishes 16 hours before transfection. Transfection was performed using 1 µg of the plasmid DNA per 30 mm dish, using JetPEI Transfection Reagent (“Polyplus–transfection”, Inc, Strasbourg, France) with N/P ratio = 7, according to the manufacturer's instructions.

### Triton solubility assay

2.5×10^5^ B^−^/B^−^ MEFs were plated on 30 mm plates 16 hours before the experiment. 24 hrs after transfection with GFP-MHC-IIB constructs, cells were washed twice with 1ml PBS and serum-starved for 24 hrs in high-glucose DMEM supplemented with 2 mM L–glutamine, 100 units/ml penicillin, 100 µg/ml streptomycin and 0.1% fatty acid-free bovine serum albumin (Sigma). After starvation, cells were lysed in 200 µl PEM buffer (100 mM PIPES pH 6.9, 1 mM MgCl_2_, 1 mM EGTA)+1% Triton-X-100+protease inhibitors mix (Sigma) for 5 minutes at 4°C. The Triton-soluble fraction was collected into fresh tubes and centrifuged for 5 min at 16,000 g to remove the remnants of the insoluble fraction. 100 µl of supernatant were transferred to fresh tubes containing 25 µl 5× SDS-PAGE sample buffer (250 mM Tris pH 6.8, 500 mM DTT, 10% SDS, 0.5% bromophenol blue, 50% glycerol) and boiled for 5 min at 100°C. Insoluble fraction was washed once with 300 µl PEM buffer and 120 µl 2× SDS-PAGE sample buffer were added (100 mM Tris pH 6.8, 200 mM DTT, 4% SDS, 0.2% bromophenol blue, 20% glycerol). Insoluble fraction was collected from dishes and boiled for 5 min at 100°C. After separation on 6% SDS-PAGE, the proteins were transferred for 4 hrs on 1A to Protran BA 85 nitrocellulose membrane using TE 62 transfer unit (Amersham Pharmacia Biotech, Piscataway, NJ). Western blotting was performed as described previously [29] using GFP rabbit polyclonal affinity purified antibody prepared in our laboratory. GFP-MHC-IIB bands were developed using EZ-ECL Chemiluminescence Detection Kit (Biological Industries) and the intensity was analyzed using FujiFilm LAS-3000 Luminescent Image Analyzer and Fujifilm ImageGauge Ver. 3.4 software (Fujifilm, Tokyo, Japan). The amount of MHC-IIB in the Triton-soluble fraction was corrected by a factor of 2 in final calculations of the percentage of MHC-IIB in that fraction.

### Microscopy

For direct fluorescence assays, 2×10^5^ cells of B^−^/B^−^ MEFs were plated on 30 mm tissue culture dishes 16 hrs before transfection. 6 hrs after transfection with GFP-MHC-IIB constructs, 0.75×10^5^ cells were re-plated on cover slips coated with 27 µg/ml collagen I (Sigma) and incubated for 16 hrs at 37°C in a humidified atmosphere. The cells were washed twice with 1 ml starvation medium (high glucose DMEM supplemented with 2 mM L–glutamine, 100 units/ml penicillin, 100 µg/ml streptomycin and 0.1% fatty acid-free bovine serum albumin (Sigma)) and serum–starved for 24 hrs in 2 ml starvation medium. After starvation, cells were washed once with 1 ml phosphate buffered saline (PBS) and fixed for 10 min in 1.5 ml 3.7% formaldehyde in PBS. After three washes with PBS, cells were permeabilized for 3 min with permeabilization buffer (PBS+0.5% BSA+0.1% Triton-X-100). After three washes with PBS, cells were incubated for 30min with 1.3 units/ml Rhodamine-Phalloidin (Molecular Probes, Eugene, Oregon). After three washes with PBS, the cover slips were mounted on slides using Vectashield mounting medium (Vector Laboratories Inc, Burlingame, CA). Cells were visualized using a 60× objective under a TE2000 inverted confocal laser scanning system (Nikon Corporation, Tokyo, Japan). Confocal images were processed using EZ-C1 software (Nikon).

### Analysis of the net charge along MHC-IIB coiled-coil

The “sliding window” technique was used to calculate the net charge. The entire MHC-IIB coiled-coil region was treated as a linear array of charged and uncharged amino acids. Values of +1 or −1 were assigned to the charged residues (lysine, arginine, positive +1; glutamic and aspartic acids, negative −1). The remaining amino acids were assigned zero charge. All charges at the **a** and **d** positions of the heptades were converted to zeros and skip residues were included. The sum of all charges in the first 98aa window was calculated. The window was then shifted by one residue towards the C-terminus end of the molecule and the net charge in the new window calculated again. This was repeated until one end of the window reached the C-terminus of the coiled-coil. The resulting data was plotted as the net charge of the window against the first residue in the same window. All calculations were carried out using Microsoft Excel, which was also used to plot the graphs in Figures 3, 7 and Figure S1.

### Analysis of the ionic interactions between adjacent molecules

Ionic interactions were analyzed by assigning +1 or −1 to a charged residue (lysine, arginine, positive +1; glutamic and aspartic acids, negative −1). Interactions of aligned charges were summed over the whole overlap region of the two chains, with unlike charges (+1 opposite −1) scoring −1 and like charges (−1 with −1 or +1 with +1) scoring +1 (i.e. charges were multiplied). Any other interactions scored 0. We assessed whether proximity of charges within, say, ±2 amino acids, should give a scored interaction. Results were not significantly altered by scoring only the exactly aligned amino acids, so this method was used. The resulting data was plotted as the net score of the interactions against the staggering between two molecules arranged in parallel (Figure 5A). All calculations were carried out using Microsoft Excel, which was also used to plot the graphs in Figure 5B.

## Supporting Information

Figure S1 Net charge distribution along the coiled-coil region of MHC-IIB. Determination of the net charge along the coiled-coil region was determined by the “sliding window” technique (see Materials and Methods). The resulting data was plotted as the net charge of the window against the first residue of the window. Note that the calculations were started from the first residue of the coiled coil (Figure 1A residue 844). The data obtained for residues 844-863 were omitted for clarity.
(0.31 MB TIF)

Figure S2 The effect of the deletion on TX-100-solubility of MHC-IIB. B-/B- MEF cells were transiently transfected with MHC-IIB or MHC-IIB mutants fused to GFP (see Materials and Methods and Figure 6A). Cells were subjected to a TX-100 solubility assay and the percentage of total MHC-IIB in the soluble fraction was determined (see Materials and Methods). The data for TX-100 solubility assay of MHC-IIB mutants are averages ± S.D of at least three independent experiments.
(0.36 MB TIF)

Figure S3 Localization of MHC-IIB mutants in polarized migrating cells. B-/B- MEF cells were transiently transfected with MHC-IIB mutants fused to GFP and subjected to a wound scratch assay with the wound at the bottom of the figure. 24hrs after inducing migration into the wound by the addition of 25ng/ml Platelet Derived Growth Factor-BB, cells were fixed and stained with rhodamine phalloidin as described in Text S1. A) Representative polarized migrating cells for each mutant are shown. Note the strong accumulation of MHC-IIB mutants in the posterior end of the cell compared to wild type MHC-IIB. Bar = 10 micrometer. B) Representative fields of confluent non-migrating cells. Note the lack of prominent posterior accumulation of deltaN197 in non-polarized cells. Only the GFP channel is shown. Bar = 20micrometer.
(9.38 MB TIF)

Text S1 (0.03 MB DOC)
