# Supplementary figures and images for: MHC-IIB Filament Assembly and Cellular Localization Are Governed by the Rod Net Charge
Source: PLoS One. 2008 Jan 30;3(1):e1496. doi: 10.1371/journal.pone.0001496 (PMC2204051; doi:10.1371/journal.pone.0001496)

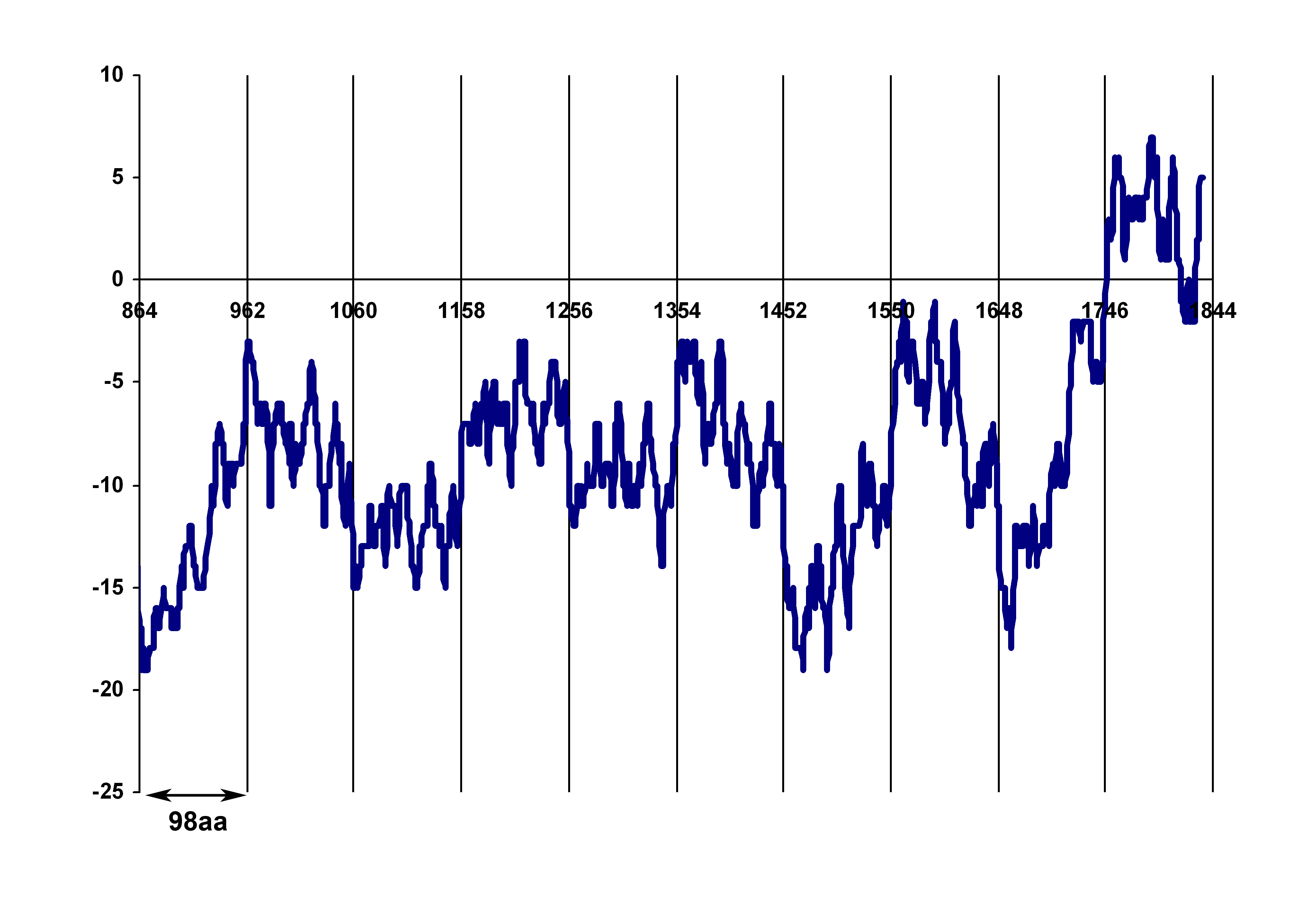

Supplement: Figure S1 — Net charge distribution along the coiled-coil region of MHC-IIB. Determination of the net charge along the coiled-coil region was determined by the “sliding window” technique (see Materials and Methods). The resulting data was plotted as the net charge of the window against the first residue of the window. Note that the calculations were started from the first residue of the coiled coil (Figure 1A residue 844). The data obtained for residues 844-863 were omitted for clarity. (0.31 MB TIF) [file pone.0001496.s001.tif]

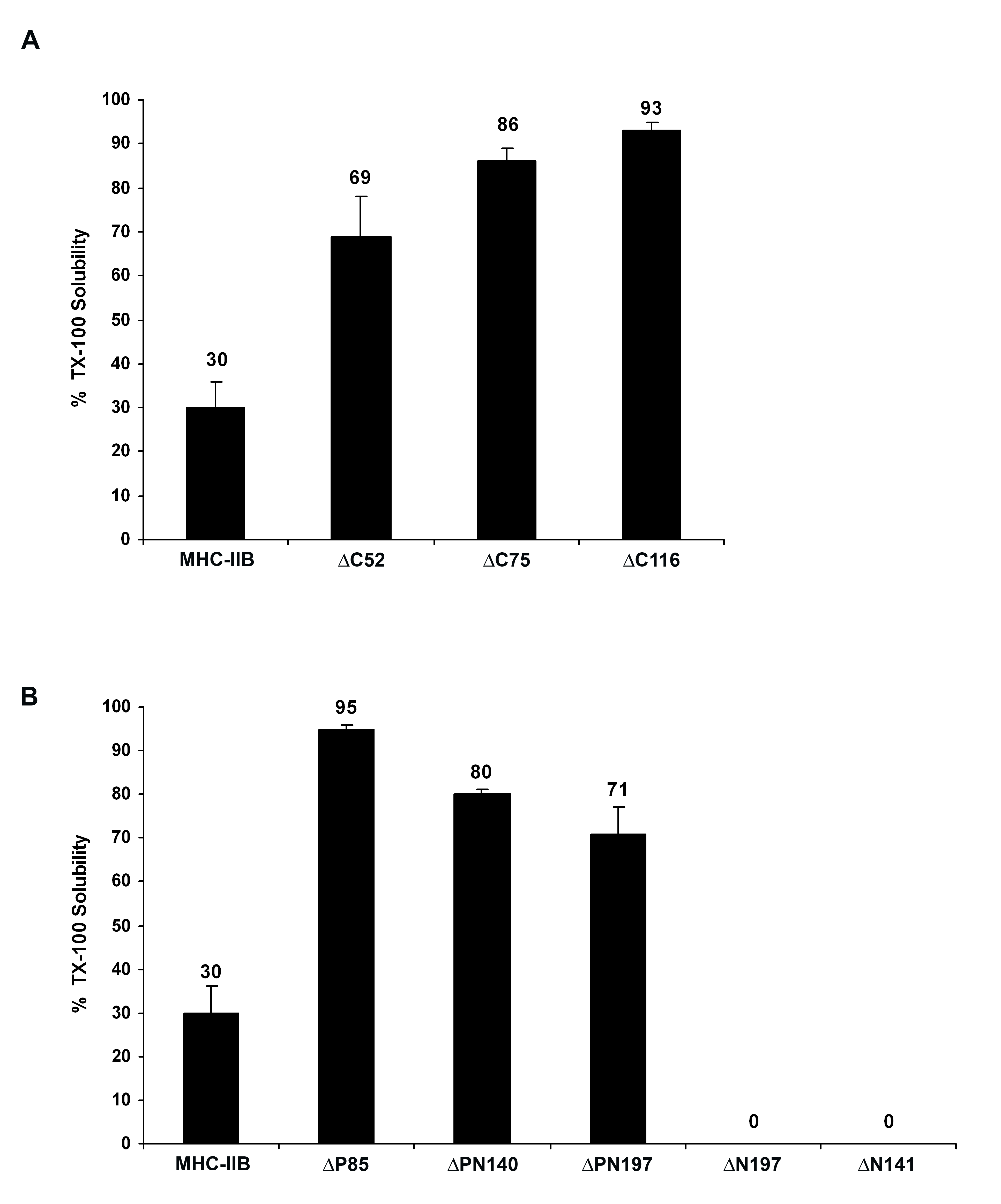

Supplement: Figure S2 — The effect of the deletion on TX-100-solubility of MHC-IIB. B-/B- MEF cells were transiently transfected with MHC-IIB or MHC-IIB mutants fused to GFP (see Materials and Methods and Figure 6A). Cells were subjected to a TX-100 solubility assay and the percentage of total MHC-IIB in the soluble fraction was determined (see Materials and Methods). The data for TX-100 solubility assay of MHC-IIB mutants are averages ± S.D of at least three independent experiments. (0.36 MB TIF) [file pone.0001496.s002.tif]

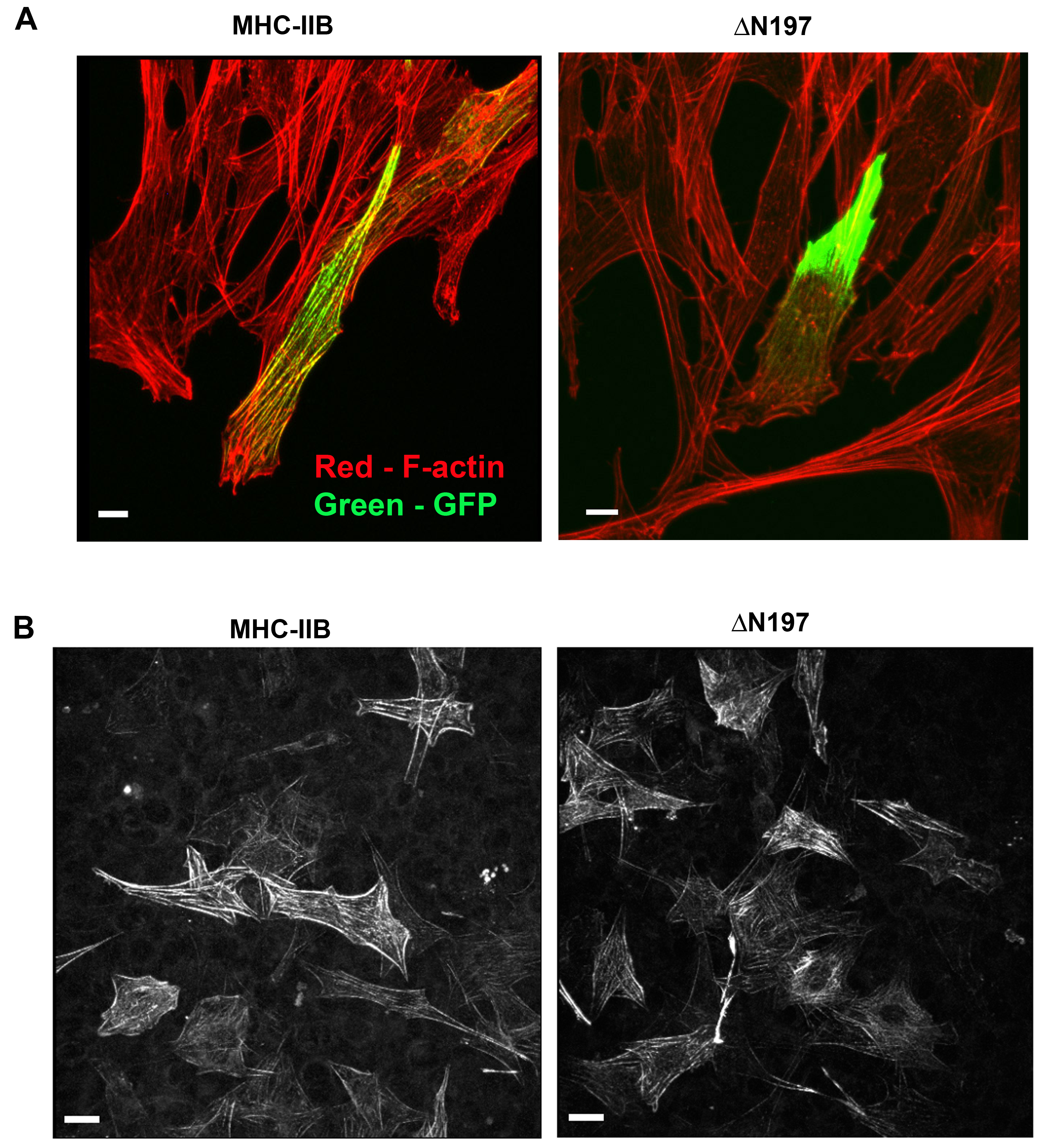

Supplement: Figure S3 — Localization of MHC-IIB mutants in polarized migrating cells. B-/B- MEF cells were transiently transfected with MHC-IIB mutants fused to GFP and subjected to a wound scratch assay with the wound at the bottom of the figure. 24hrs after inducing migration into the wound by the addition of 25ng/ml Platelet Derived Growth Factor-BB, cells were fixed and stained with rhodamine phalloidin as described in Text S1. A) Representative polarized migrating cells for each mutant are shown. Note the strong accumulation of MHC-IIB mutants in the posterior end of the cell compared to wild type MHC-IIB. Bar = 10 micrometer. B) Representative fields of confluent non-migrating cells. Note the lack of prominent posterior accumulation of deltaN197 in non-polarized cells. Only the GFP channel is shown. Bar = 20micrometer. (9.38 MB TIF) [file pone.0001496.s003.tif]
