# Supplementary material for: MHC-IIB Filament Assembly and Cellular Localization Are Governed by the Rod Net Charge
Source: PLoS One. 2008 Jan 30;3(1):e1496. doi: 10.1371/journal.pone.0001496 (PMC2204051; doi:10.1371/journal.pone.0001496)
Supplement: Text S1 — (0.03 MB DOC) [file pone.0001496.s004.doc]

### *Wound scratch assay* - 2x10^5^ cells were plated on 30mm tissue culture dishes 16hrs before transfection. 6 hrs after transfection with GFP-MHC-IIB constructs, cells were re-plated on μ-dishes (Integrated BioDiagnostics, Munchen, Germany) and incubated for 36hrs at 37^O^C in a humidified atmosphere until reaching confluence. After washing twice with 0.5ml starvation medium, cells were serum–starved for 24hrs in 800μl starvation medium followed by washes with 0.5ml starvation medium. Each μ-dish was scratched 4 times using a sterile 200μl pipette tip. After two washes with 0.5ml starvation medium, cells were stimulated with 25ng/ml platelet derived growth factor–BB (PDGF-BB) (Sigma). After 24hrs cells were washed once with 0.5ml PBS and fixed for 10min in 0.5ml of 3.7% formaldehyde in PBS. After three washes with PBS, cells were permeabilized for 3min with permeabilization buffer. After three washes with PBS, cells were incubated for 30min with 1.3units/ml Rhodamine-Phalloidin (Molecular Probes). Cells were washed twice with 0.5ml PBS and incubated in 0.8ml PBS at 40C. Cells were visualized using a 40x objective under a TE2000 inverted confocal laser scanning system and series of 0.5μm Z-stacks were created over the entire cell height. The 2-D images were created from series of Z-stacks by a maximal intensity projection tool (EZ-C1 software, Nikon).
